# A Rare Case of Chronic Coronary Artery Dissection

**DOI:** 10.7759/cureus.88099

**Published:** 2025-07-16

**Authors:** Soukaina Cherkaoui, Idriss Allalat, Mariam Lazraq, Jamila Zarzur, Mohamed Cherti

**Affiliations:** 1 Cardiology, Ibn Sina University Hospital, Mohammed V University, Rabat, MAR; 2 Cardiology B, Ibn Sina University Hospital Center, Mohammed V University, Rabat, MAR

**Keywords:** chronic coronary artery dissection, coronary artery, coronary artery angiography, coronary artery dissection (cad), young woman

## Abstract

Spontaneous coronary artery dissection (SCAD) is a rare and underrecognized cause of acute coronary syndrome (ACS), particularly in young and middle-aged women without traditional cardiovascular risk factors. While most cases heal spontaneously, a subset may persist, evolving into what can be considered a chronic coronary artery dissection (CCAD). This chronic form remains poorly defined and rarely documented.

A 44-year-old woman with a history of ST-elevation myocardial infarction (STEMI) presented several months later with recurrent chest pain. Coronary angiography revealed a persistent dissection in the mid-left anterior descending (LAD) artery without signs of active ischemia or evolving intramural hematoma. No advanced imaging was performed, but the stability of angiographic findings over time and the absence of ischemic symptoms supported the diagnosis of a chronic dissection. The initial STEMI was retrospectively considered to have resulted from an unrecognized SCAD. Given the lack of ischemia and the risks associated with intervention in SCAD, conservative management was continued. The patient remained asymptomatic and free of major cardiac events over a 12-month follow-up period.

Chronic dissection may be overlooked in patients with a history of SCAD. Although angiography remains the main diagnostic tool, it has limitations, and adjunctive imaging such as intravascular ultrasound (IVUS), optical coherence tomography (OCT), or coronary computed tomography angiography (CCTA) may help confirm chronicity. Current understanding of the long-term course of SCAD, including recurrence risk and follow-up strategies, is still limited.

CCAD should be considered in patients with recurrent chest pain and prior SCAD history. Further studies are needed to better define its prognosis, recurrence risk, and optimal monitoring strategies.

## Introduction

Chronic coronary artery dissection (CCAD) refers to the persistence of a coronary dissection weeks or months after the initial event, typically in the absence of ongoing ischemia. Although not well established in the literature, CCAD is believed to represent the later phase of spontaneous coronary artery dissection (SCAD), a rare but increasingly recognized cause of acute coronary syndrome (ACS), especially in younger women without traditional cardiovascular risk factors.

SCAD accounts for approximately 25%-35% of myocardial infarctions in women under 50 years old [1,2]. It results from an intimal tear and/or the spontaneous formation of an intramural hematoma, both of which may coexist and contribute to the dynamic obstruction of coronary blood flow. Most cases heal spontaneously, but in some instances, the dissection flap or hematoma may persist, resulting in structural changes in the vessel wall that can be visualized angiographically in the chronic phase.

Diagnosing CCAD is challenging, as angiographic findings may resemble other conditions such as recanalized thrombus, organized plaque, or fibromuscular dysplasia. Imaging modalities such as intravascular ultrasound (IVUS), optical coherence tomography (OCT), and coronary computed tomography angiography (CCTA) are helpful in both acute and chronic settings for confirming diagnosis and guiding management [3]. However, these were not performed in the present case.

Despite growing interest in SCAD, the long-term natural history, risk of recurrence, and optimal follow-up strategies, especially in chronic forms, remain poorly defined. We report the case of a 44-year-old woman with a previous ST-elevation myocardial infarction (STEMI), who presented with recurrent chest pain several months later. Coronary angiography revealed a persistent mid-left anterior descending (LAD) dissection, interpreted as a chronic phase of SCAD. This case illustrates the diagnostic complexity of CCAD and highlights the need for greater awareness of this entity in patients presenting with recurrent symptoms.

## Case presentation

A 44-year-old woman with no cardiovascular risk factors and no past medical history presented with recurrent oppressive chest pain, radiating to the left shoulder, lasting several minutes, and diminishing spontaneously. The pain occurred unpredictably, both at rest and with minimal exertion, and had worsened over the past week. She reported a similar, but more intense and prolonged episode two years earlier, diagnosed as an anterior ST-elevation myocardial infarction (STEMI) that was not explored further. Therefore, the patient did not attend routine follow-up afterward. 

**Figure 1:**
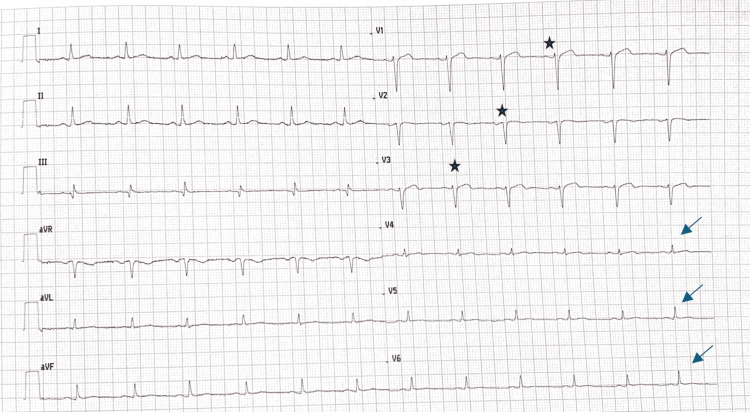
EKG showing a sinus rhythm, with a heart rate of 62 beats per minute, poor R-wave progression in the antero-septal and apical leads (black stars), and low voltage in the apico-lateral leads with biphasic T waves (arrows).

On examination, her blood pressure was 130/70 mmHg bilaterally, and her heart rate was 62 beats per minute. The cardiovascular examination was unremarkable. An electrocardiogram (EKG) revealed sinus rhythm, loss of R-wave amplitude in anteroseptal and apical leads, and low voltage in the apico-lateral leads with biphasic T waves. These findings were suggestive of prior infarction involving the anterior wall (Figure 1). Troponin levels were within the normal range.

Transthoracic echocardiography (TTE) demonstrated akinesia of the apex and adjacent segments with a preserved left ventricular ejection fraction (LVEF) of 53% (Video 1).

**Video 1:** Transthoracic echocardiography showing akinesia of the apex and adjacent segments with a preserved left ventricular ejection fraction.

**Figure 2:**
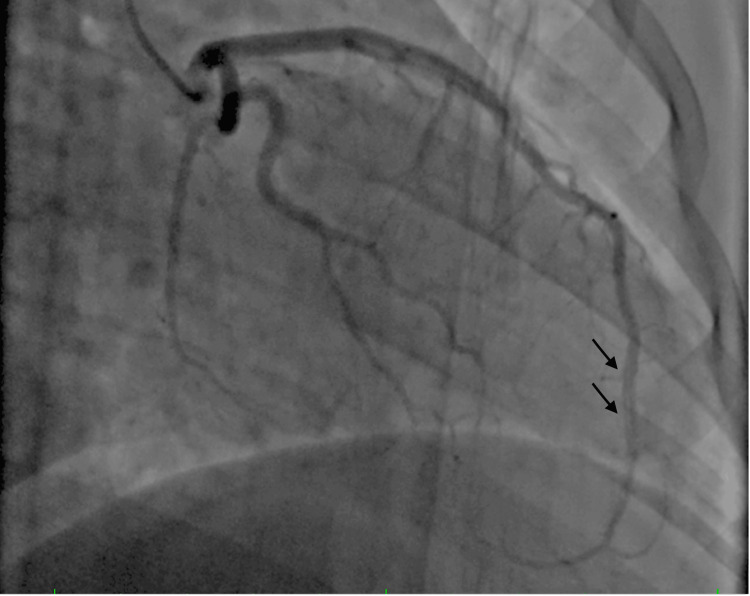
Coronary angiography showing multiple parallel channels in the left anterior descending coronary artery (arrows) likely consistent with the chronic form of type 1 dissection.

These findings are consistent with the coronary angiography results that showed multiple parallel channels in the LAD coronary artery (Figure 2).

Coronary angiography revealed a long segment of the mid-LAD artery with multiple contrast-filled linear channels, consistent with a type 1 dissection pattern. These angiographic features raised suspicion for a chronic coronary dissection. However, alternative diagnoses, such as recanalized thrombus or a woven coronary artery, could not be excluded with certainty, particularly in the absence of adjunctive imaging. IVUS or OCT was not available at the time, representing a limitation in diagnostic confirmation.

Given the preserved coronary flow (TIMI 3), absence of active ischemia, and the known risks of intervention in CAD, including propagation of dissection or iatrogenic injury, a conservative approach was adopted. The patient was treated with aspirin (75 mg daily), a beta-blocker (bisoprolol 2.5 mg daily), and an angiotensin-converting enzyme inhibitor (ramipril 5 mg daily). Following a stable clinical course, she was discharged home with planned outpatient monitoring and a six-month follow-up coronary angiogram.

**Figure 3:**
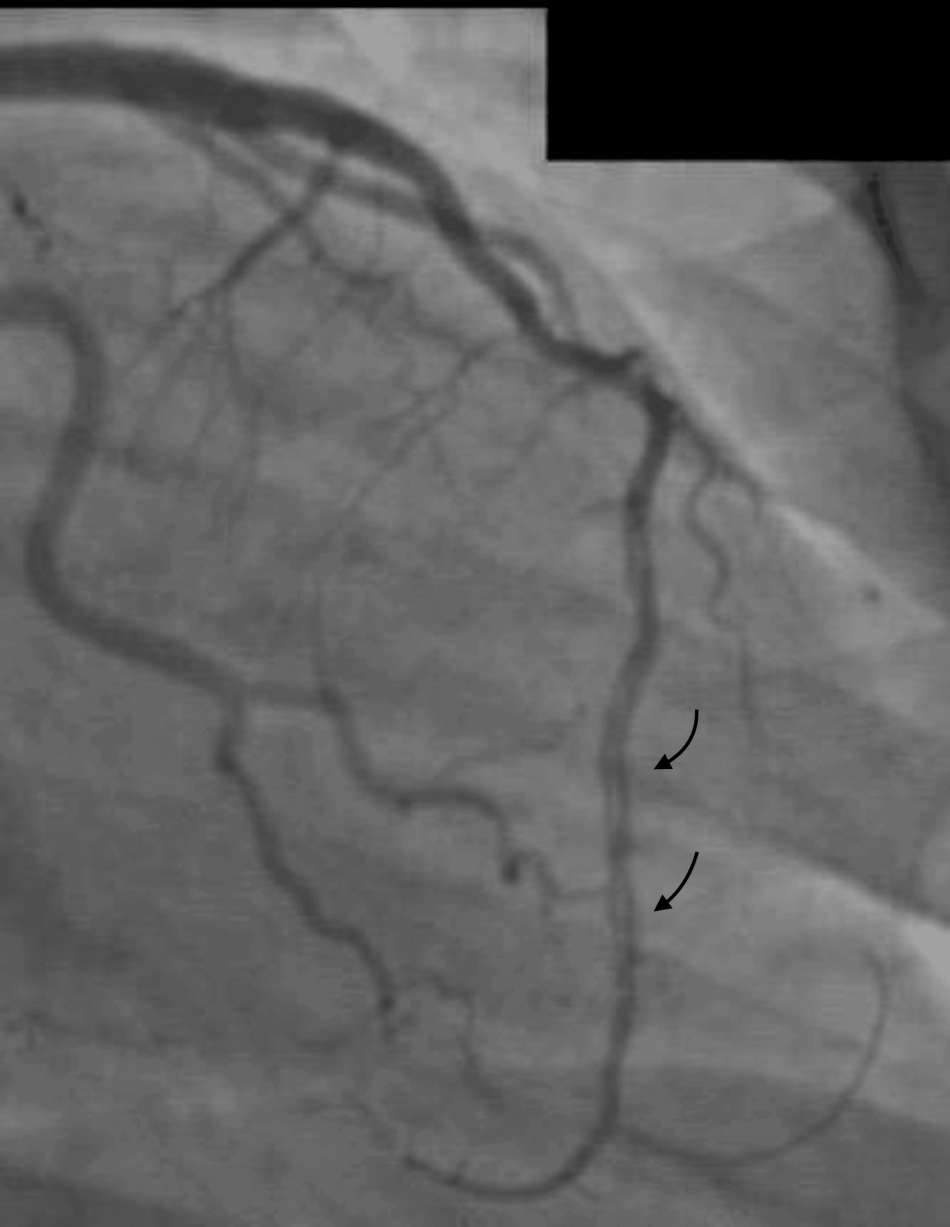
Coronary angiography control: persistent radiolucency in the distal left anterior descending coronary artery (arrows).

Despite medical therapy, the patient returned three months later with exertional chest pain that was not fully controlled. Due to symptom recurrence and its impact on daily activities, an earlier-than-planned repeat coronary angiogram was performed. The angiographic findings remained unchanged, revealing persistent dissection morphology without new stenosis or evidence of ischemia. The stability of these findings over time supports the diagnosis of a CCAD (Figure 3).

The decision was made to continue medical management, with ongoing clinical monitoring thereafter.

## Discussion

SCAD accounts for up to 4% of all ACS and is a leading cause of myocardial infarction in women under 50 years of age [1]. SCAD involves disruption of the coronary arterial wall due to an intimal tear and/or formation of an intramural hematoma, leading to compression of the true lumen and impaired blood flow [2]. Key precipitating factors include hormonal fluctuations, connective tissue disorders (e.g., fibromuscular dysplasia), pregnancy, and extreme physical or emotional stress [3,4]. The LAD artery is most frequently affected, implicated in 32%-46% of SCAD cases [2]. Imaging features suggestive of chronicity include persistent double-lumen appearance, linear filling defects, or vessel wall remodeling without signs of active hematoma progression or ischemia.

CCAD is considered a less common, late-stage manifestation of SCAD, where the dissection persists or the vessel undergoes remodeling over time, typically weeks to months after the acute event [5,6]. This may result in symptoms such as stable angina or be incidentally discovered on imaging. While no universal time-based definition of CCAD exists, chronicity is often suggested by stable imaging over time and the absence of features consistent with acute dissection, such as expanding hematoma or dynamic luminal changes.

Imaging plays a critical role in diagnosing SCAD and CCAD. Coronary angiography is the first-line tool, but its limitations must be acknowledged, particularly in differentiating dissection from atherosclerosis or thrombus. Intracoronary imaging modalities, such as OCT and IVUS, offer superior resolution for visualizing intimal disruption, intramural hematoma, or a double-lumen appearance [5,6]. CCTA may be valuable in follow-up to assess healing or chronic wall abnormalities, though its utility in acute settings is limited by motion and resolution artifacts [6].

In our case, the absence of intracoronary imaging limited diagnostic certainty. However, the angiographic appearance (multiple longitudinal contrast-filled channels within the LAD) combined with the patient’s clinical history and lack of significant atherosclerotic disease strongly supported a diagnosis of CCAD. Nevertheless, alternative diagnoses such as recanalized thrombus or woven coronary artery cannot be definitively excluded without OCT or IVUS, and this diagnostic uncertainty must be acknowledged.

The management of CCAD remains empirical and individualized, as no formal guidelines exist. In patients without ongoing ischemia or hemodynamic instability, a conservative approach is generally favored, allowing for potential spontaneous healing of the vessel wall [7]. Invasive strategies, including percutaneous coronary intervention (PCI) or coronary artery bypass grafting (CABG), are typically reserved for cases with refractory angina, high-risk anatomy, or worsening ischemia [7-9].

In our patient, despite recurrent symptoms, the dissection morphology remained stable, and there was no evidence of new ischemia or stenosis on repeat angiography. The risks associated with intervention such as propagation of dissection or procedural complications in fragile arterial segments were considered greater than the potential benefit, thus supporting a continued conservative strategy.

Long-term outcomes following SCAD are variable. Recurrence rates of 10%-30% over 10 years have been reported, although these figures are based on SCAD cohorts and cannot be directly extrapolated to CCAD due to a lack of specific data [8]. Consequently, long-term surveillance is essential and should incorporate regular clinical assessment, imaging when indicated, and education regarding symptom recognition and lifestyle modification.

Pharmacologic therapy remains empirical. Beta-blockers may reduce recurrence risk by decreasing shear stress, while low-dose aspirin is commonly used for its antiplatelet effects despite limited evidence. The role of dual antiplatelet therapy (DAPT) is more controversial, particularly in non-atherosclerotic disease where the risk of intramural bleeding must be weighed against thrombotic protection [10].

This case underscores the importance of considering CCAD in patients, especially women, presenting with recurrent chest pain and a history suggestive of prior SCAD. Further research is needed to define optimal diagnostic pathways, medical management, follow-up strategies, and prognostic implications in this underrecognized subset of coronary pathology.

## Conclusions

CCAD likely represents a late stage of SCAD and poses diagnostic and therapeutic challenges, especially when advanced imaging is unavailable. This case illustrates the limitations of relying solely on angiography, as well as the potential for symptom recurrence despite conservative management. Standardized diagnostic and follow-up protocols, along with validated risk stratification tools, are needed to guide clinical decisions and improve outcomes in this underrecognized condition.
